# An evaluation of truncated birth histories for the rapid measurement of fertility and child survival

**DOI:** 10.1186/s12963-023-00307-9

**Published:** 2023-07-18

**Authors:** Bruno Masquelier, Ashira Menashe-Oren, Georges Reniers

**Affiliations:** 1 Center for Demographic Research, UCLouvain, Louvain-la-Neuve, Belgium; 2 London School of Hygiene and Tropical Medicine, London, UK

**Keywords:** Truncated birth histories, Recall errors, Child mortality estimation, Fertility estimation, Demographic and Health Surveys

## Abstract

**Background:**

Full birth histories (FBHs) are a key tool for estimating fertility and child mortality in low- and middle-income countries, but they are lengthy to collect. This is not desirable, especially for rapid turnaround surveys that ought to be short (e.g., mobile phone surveys). To reduce the length of the interview, some surveys resort to truncated birth histories (TBHs), where questions are asked only on recent births.

**Methods:**

We used 32 Malaria Indicator Surveys that included TBHs from 18 countries in sub-Saharan Africa. Each set of TBHs was paired and compared to an overlapping set of FBHs (typically from a standard Demographic and Health Survey). We conducted a variety of data checks, including a comparison of the proportion of children reported in the reference period and a comparison of the fertility and mortality estimates.

**Results:**

Fertility and mortality estimates from TBHs are lower than those based on FBHs. These differences are driven by the omission of events and the displacement of births backward and out of the reference period.

**Conclusions:**

TBHs are prone to misreporting errors that will bias both fertility and mortality estimates. While we find a few significant associations between outcomes measured and interviewer’s characteristics, data quality markers correlate more consistently with respondent attributes, suggesting that truncation creates confusion among mothers being interviewed. Rigorous data quality checks should be put in place when collecting data through this instrument in future surveys.

**Supplementary Information:**

The online version contains supplementary material available at 10.1186/s12963-023-00307-9.

## Background

Despite recent progress, Civil Registration and Vital Statistics Systems (CRVSs) need to be complemented with surveys and censuses for monitoring mortality and fertility trends in many countries. Globally, the percentage of children under 5 years old whose birth was registered was only 65% in 2015 [1]. In 2020, 88% of under-five deaths occurred in low- and lower-middle-income countries, where retrospective surveys and censuses are often the only nationally representative data sources on child mortality [2].

In addition to the number of births that occurred over the last 12 months, censuses typically collect *summary birth histories* (SBHs), asking about the total number of children ever born to women of reproductive age and the number of children who are still alive. The proportions of deceased children are converted to life-table probabilities of dying, using the age of the mother and standard age patterns of fertility to approximate exposure to the risk of dying [3]. Fertility rates are estimated from reports on recent births that occurred in the household, usually adjusted upward to be consistent with information provided on past fertility [4]. These indirect estimates fill a critical data gap in many countries, but censuses are not frequent, at best only every 10 years. Moreover, indirect methods applied to SBHs are underpinned by strong assumptions. For example, mortality is assumed to be uncorrelated with maternal age at birth, but children from less advantaged backgrounds are overrepresented in reports from young mothers aged 15–19 years, resulting in overestimation of recent mortality [5]. Additionally, the calculation of a reference period for the estimates requires that changes in fertility and mortality have been gradual and smooth. Census estimates are therefore unsuitable for monitoring trends in contexts disrupted by conflicts or health crises such as the COVID-19 pandemic. Demographic surveys are conducted more regularly, and usually collect *full birth histories* (FBHs) with additional questions on children’s ages at the time of survey (or at the time of death), allowing for direct calculation of fertility and child mortality. Since 2020, the DHS has adopted full pregnancy histories (FPH) as the standard instrument with the intent to produce better estimates of abortions, miscarriages and stillbirths and to improve the measurement of neonatal mortality [6, 7]. However, both FBHs and FPHs are long, requiring around ten questions per pregnancy or child ever born [8]. In a study conducted in Guinea-Bissau, Ethiopia, Uganda, Bangladesh and Ghana, the mean time taken to complete a FBH and FPH was respectively 9.1 and 10.5 min [9]. This can be demanding on respondents and interviewers alike, in settings where fertility is high. Moreover, the information collected on birth or pregnancies that occurred in the distant past is of little use and more likely to be affected by recall errors. Numerous past surveys are now available to reconstruct trends, and this reduces the need for long windows of retrospection. Short birth (or pregnancy) history survey instruments are particularly desirable for new interview modalities, including telephone surveys [10], where the enumerator has less control over respondent engagement.

The *Real-Time Monitoring of Under-Five Mortality Project* tested various approaches to generate timely estimates without resorting to FBHs; the project evaluated the community-based reporting of vital events, the use of health facility data, the imputation of FBHs from an earlier survey onto SBH data from a recent survey and the tracking of cohort changes between surveys in the mean number of children ever born and surviving [11, 12]. However, these different methods did not produce acceptable estimates. Another study focusing on household reports on the survival of recent births, a short instrument for measuring mortality, showed that such reports provided mortality estimates that were biased downward and surrounded by large non-sampling uncertainty [13].

Some surveys have resorted to a different approach, *truncated birth histories *(TBHs), where questions are asked only on recent births. Recent is defined in relation to the date of the interview (e.g., last 5 years) or in terms of the number of births (e.g., last two or three births). TBHs have been employed in most Malaria Indicator Surveys (MISs) organized by the Demographic and Health Surveys (DHSs) program. Some Reproductive Health Surveys (RHSs), Contraceptive Prevalence Surveys and specific surveys focused on child mortality also included a TBH [14]. The Performance Monitoring and Accountability 2020 (PMA2020) surveys collected the details for the last two births [15].

TBHs are appealing for rapid evaluation of fertility and child mortality, but they have several disadvantages. First, TBHs offer fewer opportunities to reconstruct trends, or, to conduct analyses by cohort and birth order. Second, mortality in older children and young adolescents aged 5–14 years cannot be estimated from TBHs, and an important proportion of premature mortality takes place at these ages [16]. Third, the scope for data quality assessments is more limited, while reporting errors and selection biases could be more pervasive than in FBHs. Respondents or interviewers may be tempted to shift some births into the past to locate them beyond the truncation date in order to save time, a problem already identified in FBHs to avoid additional lengthy sections of the questionnaire on nutrition and health of recently born children [17]. There could also be genuine difficulties in placing births in the reference period. Additionally, the omission of children, especially deceased children, might be more frequent because interviewers cannot check the consistency of information provided in the TBH against summary data on the total number of children ever born and surviving, which are usually collected as precursor. Fourth, last-born children are overrepresented in TBHs, and this could introduce selection bias in the mortality estimates, as birth order is often associated with mortality [18].

Few studies have examined recall and selection biases in TBHs, with inconclusive results. TBHs were first tested in 1986 in DHS conducted in Peru and Dominican Republic [19, 20]. In both countries, an experimental survey was conducted using TBHs in a nationally representative sample, while a larger sample was surveyed with the standard DHS questionnaire. There were no significant variations in age-specific fertility rates or child mortality for the recent period (1980–86) between the FBH and TBH datasets, with no clear evidence of omissions or displacement of births. The authors concluded that “the type of truncated history incorporated into the experimental questionnaire appears to be an efficient and reliable data collection strategy” [19]. Fertility indicators were also comparable across types of questionnaires in the Dominican Republic surveys [20]. In contrast, a comparison of the 1998 RHS in Mongolia, based on a full birth history, with the 2003 and 2008 RHS based on TBHs, showed compelling evidence that deceased children were disproportionately omitted or transferred out of the reference period in the TBHs [21].

Given the use of TBHs in some survey programs and their potential to reduce time, costs as well as interviewer and respondent fatigue in future surveys, a more systematic evaluation is needed. In this study, we analyze 32 Malaria Indicator Surveys (MIS) from 18 countries in Sub-Saharan Africa in which TBHs were collected. Each survey is paired, and compared, to at least one survey that collected FBHs in the same country around the same period. We consider conventional data checks, such as the sex ratio at birth or the share of children reported in the recent past, and estimate fertility and mortality using the available data. Finally, we evaluate whether mortality indices obtained from TBHs could be affected by birth-order compositional effects.

## Methods

We use 32 TBHs conducted as part of the Malaria Indicator Surveys between 2006 and 2021 in 18 countries, all in sub-Saharan Africa, and pair them up with surveys that collected FBHs. All but one survey with TBHs (Angola 2006–7) are paired up to a preceding survey with FBHs, while 14 surveys are also paired up to a survey collected in the following years. In total, our sample is made up of 45 pairs of surveys. Since some TBH surveys are represented twice (paired with both preceding and following surveys), we test for sensitivity of our results by only using preceding pairs. Our comparator is most often a standard DHS survey, but in six cases, we match a MIS that collected TBHs with another MIS that collected FBHs. Matched MIS likely reduce differences across surveys like in interviewer training or survey duration. In addition to the Peru and Dominican Republic surveys mentioned above [19, 20], and surveys conducted in El Salvador (1985) and Nigeria (Ondo State, 1986), which cannot be matched to a FBH in comparable period, these 32 MIS surveys are the only ones to have used a TBH within the DHS program. The MICS surveys did not collect TBHs, and we know only of three TBH datasets available in the public domain from the RHS program. We limit the analysis to MIS surveys to work with a coherent set of surveys with similar content, duration and interviewer training.

**Fig. 1:**
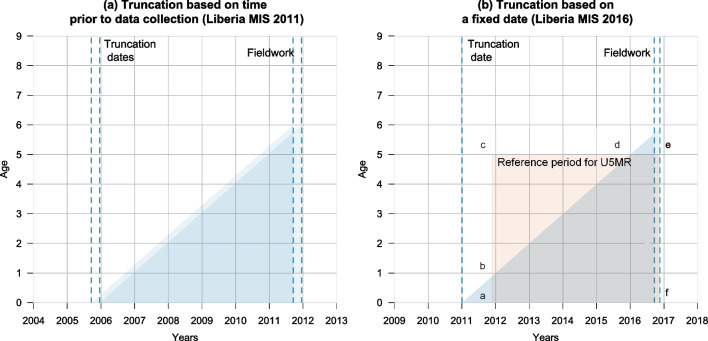
Illustration of the truncation in MIS surveys conducted in Liberia in 2011 and 2016

Table 1 lists all survey pairs and includes information about the truncation period in TBHs, as indicated in the questionnaires. Henceforth, we refer to MIS that used a TBH as “TBH surveys”, and to the standard DHS or MIS surveys based on a FBH as “FBH surveys”. We refer to the period between the truncation date and the survey date as the “reference period”. Ten TBH surveys define this period as a fixed interval before data collection, and the reference period is the same for all respondents (6 years^1^) (Fig. 1a). Twenty-two surveys use a calendar date as the cutoff (e.g., since 2016) and the length of the reference period will vary across surveys and individuals (Fig. 1b). The average length of the reference period varies from 5.4 to 7.0 years in the TBH surveys.

**Fig. 2:**
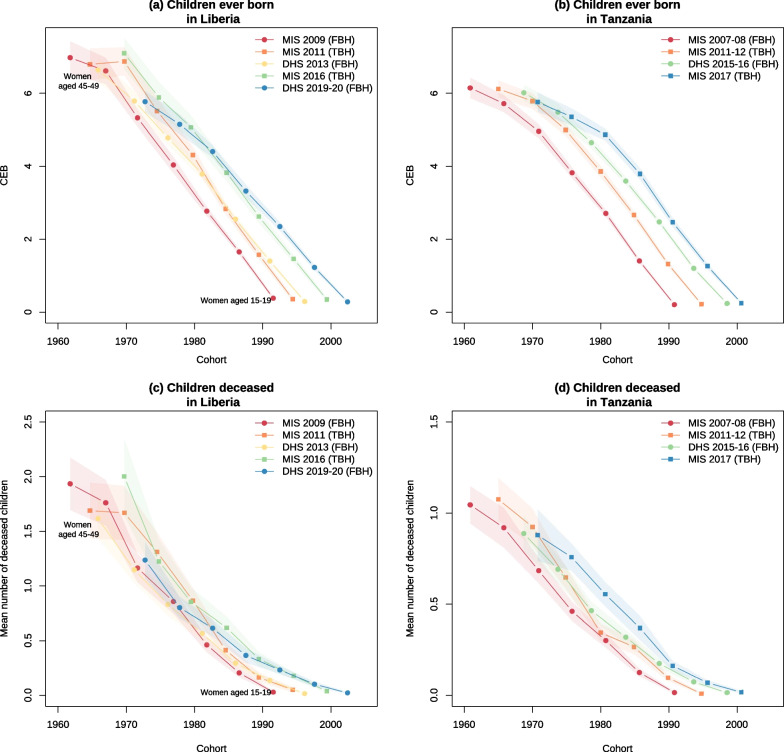
Mean number of reported children ever born and deceased in Liberia and Tanzania surveys *Note* Squares correspond to estimates obtained from TBH surveys, while circles refer to FBH surveys

Both TBHs and FBHs are preceded by a summary birth history; an enumeration of children living with their mothers, those living elsewhere and those deceased. All TBH surveys then collected data on the sex of recently born children, their type of birth (single or multiple), their date of birth, their survival status, their current age when surviving, whether they lived with their mother and their household line number for those living in the household, starting with the most recent birth. In the surveys they are paired with, FBHs have a similar structure, but the data are entered in the reverse order, from the first birth to the last, and systematically include an additional question on ages at deaths.

**Table 1:** Surveys included in analysis

Country	TBH survey	Truncation	Preceding FBH survey	Following FBH survey
Angola	2006–07 MIS	“Since 2001”		2011 MIS
Burkina Faso	2014 MIS	“Since 2008”	2010 DHS	
	**2017–18 MIS**	“2012–2017”	2010 DHS	
Burundi	2012 MIS	“from January 2006”	2010 DHS	2016–17 DHS
Ghana	**2016 MIS**	“2011–2016”	2014 DHS	
	**2019 MIS**	“2014–2019”	2014 DHS	
Guinea	**2021 MIS**	“2016–2021”	**2018 DHS**	
Kenya	**2015 MIS**	“From January 2010”	2014 DHS	
	**2020 MIS**	“2015–2020”	2014 DHS	
Liberia	2011 MIS	“Last 6 years”	2009 MIS	2013 DHS
	**2016 MIS**	“2011–2016”	2013 DHS	**2019–20 DHS**
Madagascar	2011 MIS	“Last 6 years”	2008–09 DHS	
	2013 MIS	“Last 6 years”	2008–09 DHS	
	2016 MIS	“2011–2016”	2008–09 DHS	
Malawi	2012 MIS	“Last 6 years”	2010 DHS	**2015–16 DHS**
	2014 MIS	“Last 6 years”	2010 DHS	**2015–16 DHS**
	**2017 MIS**	“2012–2017”	**2015–2016 DHS**	
Mali	2015 MIS	“Since 2009”	2012–13 DHS	2018 DHS
	**2021 MIS**	“2016–2021”	**2018 DHS**	
Mozambique	**2015 AIS/MIS**	“From January 2009”	2011 DHS	
	2018 MIS	“2013–2018”	2011 DHS	
Nigeria	**2015 MIS**	“Last 6 years”	2010 MIS	**2018 DHS**
Rwanda	2013 MIS	“Last 6 years”	2010 DHS	2014–15 DHS
	2017 MIS	“2012–2017”	2014–15 DHS	**2019–20 DHS**
Senegal	2006 MIS	“Last 6 years”	2005 DHS	2008–9 MIS
	**2020–2021 MIS**	“2015–2020”	2019 DHS	
Sierra Leone	**2016 MIS**	“2011–2016”	2013 DHS	2019 DHS
Tanzania	2011–12 AIS/MIS	“Last 6 years”	2007–08 MIS/AIS	2015–16 DHS
	**2017 MIS**	“2012–2017”	2015–16 DHS	
Togo	2017 MIS	“2012–2017”	2013–14 DHS	
Uganda	2014–15 MIS	“Last 6 years”	2009 MIS	**2016 DHS**
	**2018–19 MIS**	“Since 2013”	**2016 DHS**	

Surveys for which we use data on interviewer characteristics are in bold. In the 2013 MIS in Madagascar, the cities of Antananarivo Renivohitra, Antsirabe I and Fianarantsoa I as well as municipalities with an altitude higher than 1500 m were excluded from the sample as the malaria endemicity is very low in these areas. It is therefore not strictly comparable to the 2008–9 DHS. Three Northern regions in Mali (Gao, Tombouctou and Kidal) were not surveyed in the 2015 MIS (due to security issues), so these regions were similarly excluded from our analysis in the DHS 2018 (when compared to the 2015 MIS). They were not part of the 2012–2013 DHS sample. These regions were included in the sample of the 2021 MIS

For the 6 pairs of surveys that compare two MIS surveys, differences in data quality can be directly attributed to the truncation of the birth histories. For the other 39 pairs of surveys, other sections of the questionnaire will differ, and this may have repercussions for the quality of the birth history data. In the MIS, about 20 additional questions are asked for each child listed in the birth history about the frequency of fevers, use of care and treatment. In the standard DHS, follow-up questions are asked for children born in the last 5 years, on fertility intentions at the time of pregnancy, pre- and postnatal care, delivery assistance, vaccinations, nutrition, prevalence of fevers and diarrhea and care given in case of infection. (Some questions are only for the last birth or two.) Incentives for interviewers to displace births are thus present in both types of surveys. Gains in terms of workload reduction could be greater in the case of displacement or omission of a birth in standard surveys than in MIS. Previous analyses suggest however that such displacements are rare in standard DHS surveys (approximately 2% for births and 5% for deaths) [8].

On average, the time difference between the TBH and the paired FBH surveys is 3.2 years (median of 3.0 years), with a maximum of 7.3 years difference and a minimum of 1.0 year. The majority of TBH surveys are compared to FBH surveys from earlier years. As such, slightly lower fertility or mortality should be captured in the TBHs than in the FBHs. As the TBH surveys have a smaller sample size than the corresponding FBH surveys (an average of 6600 women interviewed in TBH surveys versus 14,300 in FBH surveys we examine), uncertainty around estimates derived from TBHs will be larger.

### Sources of bias and checks employed

All birth history estimates of fertility and mortality rest on the assumptions that reporting on live and dead children is similarly accurate, that dates of birth and ages at death are reasonably accurately reported, and that there is no correlation between the mortality risk of a child and the survival of mother. Bias in estimates also arise when dead mothers had different fertility than surviving mothers [22, 23]. Several studies have been conducted on the quality of birth histories in DHS and we build on this work for examining TBH surveys [8, 17, 24]. Unfortunately, in the majority of the TBH surveys, information on age at death was not collected, meaning that some checks are only possible in two countries, Angola and Mozambique.

First, we examine the mean number of children ever born and deceased, irrespective of the timing of births. This is useful to establish whether the summary birth histories are complete before examining the fraction of children reported after the truncation date. We compare the mean numbers of children ever born and deceased by the mothers’ birth cohort. For example, the average parities reported by women aged 35–39 years in a survey conducted in 2020 should be higher than the average among women aged 30–34 years in a survey conducted 5 years earlier. A decline in the average parities as we follow each cohort through time reveals evidence of omission of children. We also consider whether female births are more likely to be omitted by examining sex ratios at birth.

Second, we estimate mortality indirectly, using the proportion dead among all children ever born classified by age group of mothers [21]. We use coefficients from the North pattern of Princeton life tables to estimate life table survivorship probabilities and convert all age-specific values to the under-five mortality rate, as this pattern is most appropriate for sub-Saharan Africa [25]. Again, these estimates are based on summary data collected before the TBHs or FBHs, so we do not expect deviations between the instruments, unless there are systematic differences in sample designs, interviewer’s training and conduct or respondent fatigue.

**Fig. 3:**
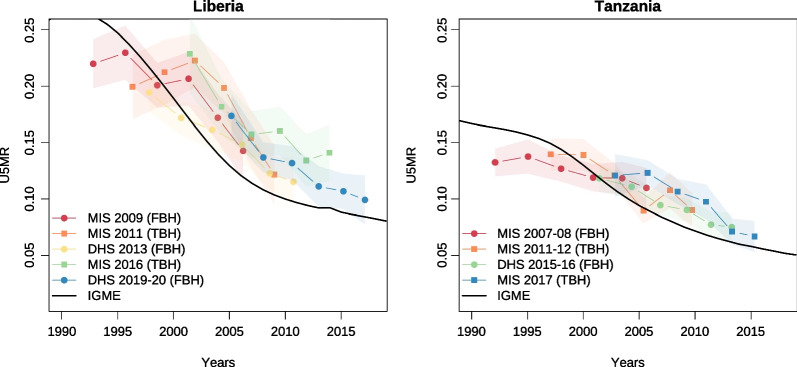
Trends in under-five mortality as estimated indirectly in Liberia and Tanzania and as estimated by UN IGME (2022) *Note* Indirect estimates are derived from information provided by mothers aged 20–49 years. Squares correspond to estimates obtained from TBH surveys, while circles refer to FBH surveys

**Fig. 4:**
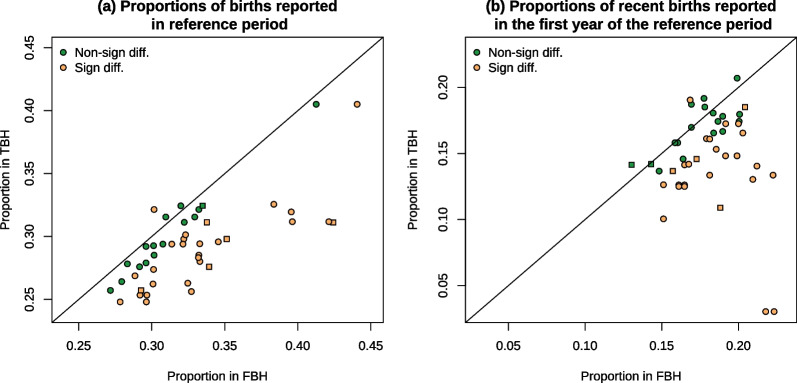
**a** Proportions of all lifetime births that are reported in the reference period and **b** Proportions of all recent births (in the reference period) that are reported in the year immediately following the truncation date *Note* For surveys that used a fixed truncation date (e.g., January 2011), we calculated the proportion of births reported in 2011 among those reported in the first 5 years of the reference period, to account for the fact that the last calendar year is not complete. We do the same for FBHs. Survey pairs associating two MIS surveys are identified with squares

Third, we turn to the proportion of children born before or after the truncation date. In FBH surveys, we replicate the truncation by considering only children born in the 6 years before data collection or using the mean length of the reference period of the paired TBH survey if defined based on a calendar year. The percentage of recent births is expected to be similar, as surveys were conducted close enough in time. We compute the total fertility rates (TFRs) in the reference period to assess whether there are missing children during this period. We compare TFR values with World Population Prospects (WPP) estimates [26], which are compiled from all available national sources and constructed to be consistent with other components of population dynamics. The WPP estimates may themselves be subject to error as they are informed by imperfect survey and census data in the set of countries considered here [27]. However, in the absence of exhaustive systems of vital registration, they provide the best possible comparator, based on standardized and transparent methods.

Fourth, we examine the proportion of children who died before and after the truncation period. If children who died in the reference period are likely to be omitted or have their birth dates moved back so that they do not appear in the truncated birth history, then the proportion of children who died in the reference period will be too low compared to FBH surveys, and the proportion dead before the truncation date will be too high [21]. In Angola and Mozambique, we can directly compare the age-specific risks of dying between TBH and FBH surveys.

Fifth, we evaluate the quality of reporting of dates of birth. Because TBHs collect data over a shorter period of time, higher quality of data on birth dates is expected. We compute the percentage of children born in the reference period for whom imputation was required to establish the date of birth because the day, month and/or year was missing.

We further examine whether the variations observed between TBH and FBH surveys are associated with characteristics of the women interviewed, or, of the enumerators. This is possible for a subset of surveys where data on interviewer characteristics were collected. (Those surveys are identified in bold in Table 1) [24]. We use linear regression models to predict the reported number of children born alive and deceased. We included in the models the women’s age group, educational attainment and place of residence, in addition to the sex of interviewers, the highest level of education they attained, whether they have themselves experienced any child death as parents and whether they had previously worked for the DHS Program (for standard DHS or MIS surveys). Because the number of interviewers can be quite small (100 interviewers on average in TBH surveys analyzed here), and interviewers are usually assigned to one region or two, interpreting the results per survey can be challenging. Hence, we also evaluate these associations in datasets where we pooled all TBH or FBH together, and include, in addition to the covariates listed above, survey-specific fixed effects. Before pooling the datasets, we de-normalized the survey weights so that each dataset had an equal weight [28]. We run logistic regressions predicting the proportion of births or deaths reported in the reference period, using the same set of covariates, in datasets from each survey and the pooled datasets.

Finally, we assess the magnitude of biases associated with the truncation. Births reported in TBH are not representative of all births contributing to the mortality experience of children aged 0–5 years in the recent period. As observed in Fig. 1b, children reported in a TBH only contribute to the bottom triangle in the Lexis diagram (more precisely, the area noted *abdef*). But under-five mortality is usually calculated for periods, most often for the full 5-year period prior to the survey (the square labelled *acef*). Some children contribute to the calculation of exposure times and deaths for this reference period in the case of a FBH, but they do not appear in a TBH. This poses three potential problems. First, the mortality experience of cohorts that do not appear in the calculation (the *bcd* triangle) could differ from the experience of those born recently, for example, because of differences in the composition of these subsamples by maternal age or birth order. The second problem is that the estimation of mortality at older ages is based on only a small number of cohorts; in our example, children 4 years of age will only contribute person-years and deaths in the last 2 years. Mortality rates at age four will need to be approximated by rates measured in recent years when estimating under-five mortality over the last 5 years. If mortality has declined over time, this assumption will result in downward bias. The third problem is that losing the *bcd* triangle in Fig. 1b increases the uncertainty around the mortality levels calculated for reference periods longer than 2 years, because the calculation is based on fewer person-years of exposure. To evaluate the magnitude of truncation bias, we extract under-five mortality rates from FBH surveys based only on the recent births (the *abdef* area) and compare them with the estimates obtained conventionally from all births.

We use the survey package of the R statistical software [29] to take into account the stratified two-stage cluster design of DHS surveys for all calculations and regressions. Standard errors around mortality and fertility rates are obtained using a Jackknife variance estimation method [30].

## Results

### Mean number of children ever born and deceased

The upper panel of Fig. 2 presents the mean number of children ever born in successive surveys in Liberia and Tanzania, two countries selected because they each have a MIS survey with TBHs and a MIS with FBHs. The estimates are derived from the SBHs. The x-axis refers to women’s birth cohort, so the curves of successive surveys should follow each other without crossovers. In Liberia, the values reported in the three MIS are quite consistent, irrespective of the type of birth histories collected afterward, with a gradual rise in the mean number of children ever born. In contrast, the curves derived from the standard 2013 and 2019–2020 surveys intersect those from several previous surveys, suggesting that older women omitted some of their children. This pattern is observed in several countries (see Additional file 1: Fig. S1 for all survey pairs). Surveys are more consistent in Tanzania, and again the two TBH surveys do not seem to be affected by disproportionate underreporting, except perhaps among older women in the 2017 MIS. Across all survey pairs, the mean number of children ever born reported in TBH surveys tend to be higher than those reported in the preceding FBH surveys, as it should be. In the few cases where they are lower (only 3% of the age-specific estimates), these differences are not significant. In sum, there is no sign of a disproportionately large number of omissions in the SBHs collected in the TBH surveys compared to the FBH surveys. In contrast, when comparing the parities reported in the TBH surveys with the FBH surveys that follow, crossovers are observed in 9 of the 14 survey pairs, and in two pairs, these differences are statistically significant. This is a sign of underreporting of children in FBH surveys, most of which were standard DHS with longer questionnaires than the TBH surveys they are compared to. We found no significant difference between survey pairs in the gender distribution of children ever born.

**Table 2:** Direct estimates of risks of dying in childhood in the Angola 2006–7 MIS and Mozambique 2015 AIS and in the paired surveys, UN IGME 2022 estimates for the mid-point of each reference period

	Angola		Mozambique	
	MIS 2006–7 (TBH)	MIS 2011 (FBH)	2015 AIS (TBH)	2011 DHS (FBH)
Neonatal mortality	23.8 (14.3–33.2)	23.3 (19.1–27.4)	14.0 (9.9–18.2)	30.4 (26.0–34.7)
Infant mortality	68.4 (55.6–81.1)	48.7 (42.8–54.7)	25.0 (19.7–30.4)	63.4 (57.0–69.8)
Under-five mortality	94.6 (73.1–115.5)	84.3 (72.2–96.2)	43.1 (33.1–53.0)	86.8 (76.7–96.8)
*Reference period*				
Neonatal/infant mortality	2004.5	2008.7	2013.0	2009.1
Under-five mortality	2006.0	2010.2	2014.5	2010.6
*UN IGME estimates*				
Neonatal mortality	44.4 (35.6–54.7)	38.2 (27.4–52.0)	32.4 (23.8–42.5)	34.7 (27.0–42.9)
Infant mortality	103.2 (88.9–119.9)	82.1 (63.5–105.1)	63.4 (54.9–73.4)	71.5 (66.0–77.5)
Under-five mortality	170.4 (143.8–201.5)	131.5 (98.2–174.0)	92.1 (77.8–109.2)	107.1 (97.7–117.5)

The lower panel of Fig. 2 compares the mean numbers of deceased children. Again, crossovers are a sign of poor data quality as the mean number of deceased children should only rise over time within cohorts. We observe such crossovers in Liberia and Tanzania, but the differences across surveys are not significant. When considering all TBH surveys and comparing these to preceding FBH surveys, there is only one survey pair with a significant difference (Additional file 1: Fig. S2). By contrast, when comparing TBH surveys with those that followed, we found four FBH surveys with a significantly lower mean number of children deceased than reported in the paired survey, pointing again to some omissions of children (see the Liberia DHS 2013 survey compared to the MIS in 2011 in Fig. 2c).

### Indirect estimates of under-five mortality

Indirect mortality estimates confirm the good quality of the summary data on children ever born and deceased in TBH surveys. Figure 3 compares the under-five mortality rates (U5MR) from the TBH surveys in Liberia and Tanzania, with the paired FBH surveys, alongside the trend reconstructed by the UN Inter-agency Group for Child Mortality Estimation [31]. The UN IGME estimates are partly based on the MIS (indirect) and DHS (direct) estimates, but they also rely on many other surveys and censuses and account for recall errors through statistical modeling [2]. Most surveys provide higher levels of U5MR than the UN IGME trend, irrespective of the type of questionnaire used (Additional file 1: Fig. S3). The median ratio of the indirect U5MR over the UN IGME level is 1.15 in the 32 TBH surveys, but only 1.02 in the subset of matched surveys that used a FBH. This slight overestimation of mortality with the indirect approach has been observed previously and can be related to violations of the assumptions of the method rather than recall errors [32]. In any case, these ratios higher than 1.00 confirm that TBH surveys are not disproportionately affected by underreporting of live born or dead children, when considering the summary data collected beforehand.

### Timing of births and fertility rates

A different pattern emerges when considering the timing of births and fertility rates. In 28 of the 45 survey pairs, the proportion of children reported as being born during the reference period was significantly lower in the TBHs than in the matched FBHs, while the reverse was true in only one survey pair (Fig. 4a). On average, 10% of recent births were missing in TBHs. This percentage was similar when restricting the analysis to survey pairs in which the FBHs came from a preceding survey (11%).

**Fig. 5:**
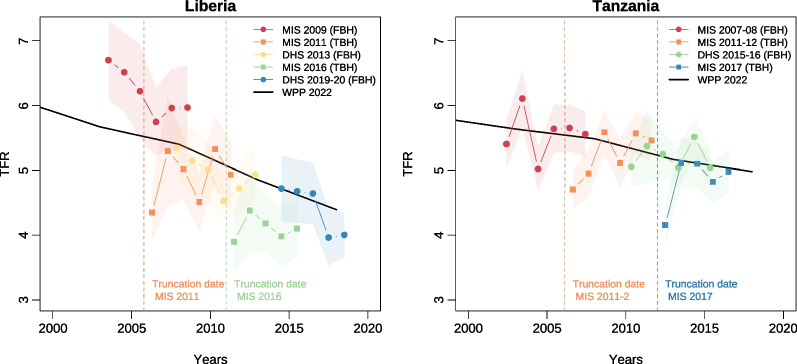
Trends in total fertility as estimated from surveys in Liberia and Tanzania and in the World Population Prospects 2022. *Note* Squares correspond to estimates obtained from TBH surveys, while circles refer to FBH surveys

**Fig. 6:**
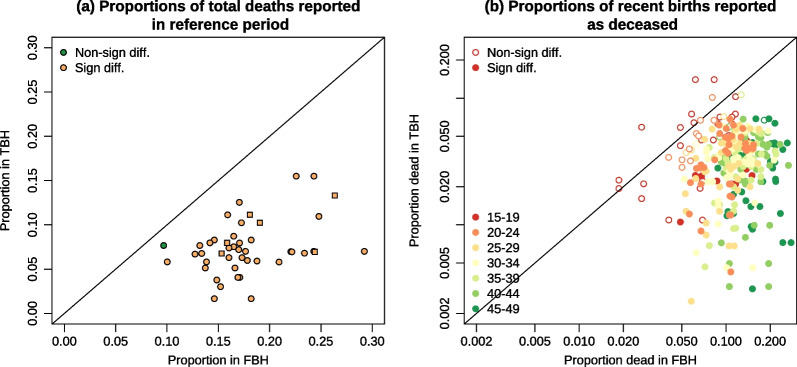
**a** Proportion of deceased children reported in reference period and **b** proportion deceased among births in the reference period, all survey pairs. *Note* Survey pairs associating two MIS surveys are identified with squares

**Fig. 7:**
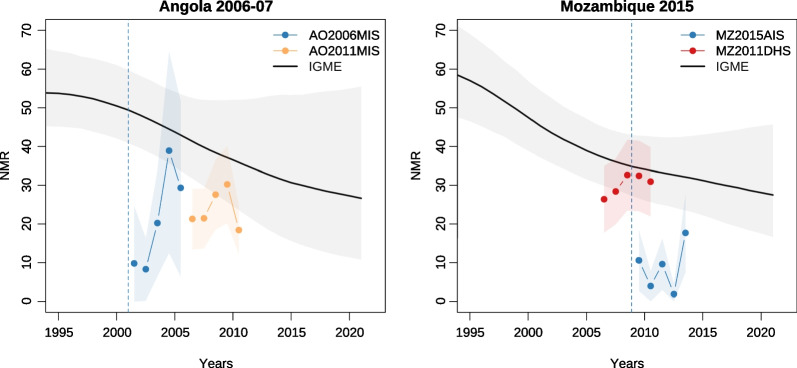
Trends in neonatal mortality in Angola and Mozambique from TBH and FBH surveys, and estimates from UN IGME (2022). *Note* Shaded areas around the survey estimates refer to 95% CI, while the shaded areas around the IGME estimates refer to 90% CI

**Fig. 8:**
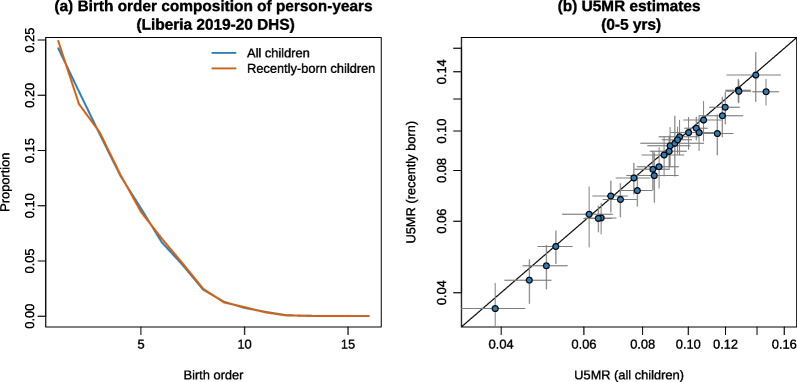
**a** Birth order composition of the person-years for all children reported in the Liberia 2019–20 DHS and only for children born in the last 5 years, **b** U5MR estimates obtained from the full sample and the truncated sample (horizontal and vertical lines refer to the 95% CI around the estimates)

An examination of the proportion of births reported in the first year of the reference period suggests that these births are more likely to be missed or displaced (Fig. 4b). Among births reported in the reference period, the proportion reported in the first year is 16% lower in TBHs compared to FBHs. With the available data, it is impossible to determine whether these lower proportions are due to omissions, or, transfers of children out of the reference period.

Such omissions and transfers result in biased fertility rates. Figure 5 presents the total fertility rate (TFR) per annum and compares them to WPP estimates in Liberia and Tanzania. There is relatively good agreement between the TFR values between surveys, which are also consistent with the 2022 WPP estimates. Note, however, that in each TBH survey, the value closest to the truncation date shown in the dashed lines is considerably lower than the more recent estimates. This pattern is observed in many other TBH surveys (see Additional file 1: Fig. S4). The median ratio between the TBH TFRs and the WPP estimates is 0.99 when considering the year closest to the data collection, but this median ratio drops to 0.78 when approaching the truncation date. With FBHs, the median ratio is 1.00 for the year before the survey and 0.96 for the year immediately after the truncation date. These results confirm that a sizeable proportion of children born near the truncation date were not mentioned in the TBH surveys.

### Timing of deaths and mortality rates

Children missing from TBHs are more likely to be deceased children. Figure 6a shows the proportion of total deaths reported in the reference period, and points to massive underreporting of deceased children, even though they may have been reported when women were asked about lifetime fertility. Of all the deaths initially reported in response to the summary questions, 18% were attributed to the reference period in FBH surveys against only 7% in the TBH surveys. In more than half of all survey pairs, the proportion of deaths that were in the reference period was at least twice higher in the FBH compared to the TBH.

Figure 6b provides another perspective and displays the proportion of deceased children among recent births, by maternal age. The median ratio of the proportion of deceased children among recent births in FBHs to the same proportion in TBHs is 3.6. Underreporting of deceased children increases with mother’s age; the median ratio rises from 2.2 in women aged 15–19 years to 6.2 in women aged 45–49 years.

In Angola and Mozambique, we can estimate mortality rates directly based on the TBHs, as data on ages at death were collected (Table 2). To account for the truncation, we restrict the estimation of under-five mortality to the last 2 completed years before the survey, while neonatal and infant mortality are estimated for the last 5 completed years before data collection. The last three rows of Table 2 refer to estimates from the UN IGME, interpolated to obtain a value for the midpoint of each reference period [31]. In Angola, the age-specific mortality rates estimated from the 2006 MIS, which collected TBHs, are close to those inferred from the 2011 MIS, based on FBHs. Confidence intervals around the estimates overlap for neonatal and under-five mortality, and the infant mortality rate is higher in the 2006 survey. This could reflect real trends in mortality. The quality of the 2011 MIS itself can however be questioned. The mean numbers of children ever born and deceased are too low (see Additional file 1: Figs. S1, S2), and the indirect estimates are also much lower than estimated by the UN IGME (Additional file 1: Fig. S3). When comparing values from the 2006–7 MIS or 2011 MIS with the IGME estimates, the latter are significantly higher. The neonatal mortality rates (NMR) estimated in the 2006–7 and 2011 MIS are one-half and two-thirds, respectively, of the rates estimated by the UN IGME for the same periods. We conclude that the Angola 2011 MIS is not of sufficiently high quality to serve as a baseline for evaluating the TBH data collected in 2006–7.

In Mozambique, the 2011 FBH survey serving as a reference appears to be of better quality. Fertility rates derived from this survey are consistent with the WPP estimates (Additional file 1: Fig. S4), and mortality levels calculated directly are consistent with the UN IGME values for the same reference periods. This is of course partly because this 2011 survey informed the UN IGME trend, but other data sources were also exploited (e.g., 2008 MICS, 2015 AIS, 2017 census, 2018 MIS) [2]. Using this 2011 survey as a reference, we conclude that the TBH data collected in 2015 are afflicted by substantial underestimation. The neonatal and under-five mortality rates are about half the levels estimated via the 2011 DHS 4 years earlier, while infant mortality rates are nearly one-third the level inferred from the 2011 survey. An examination of annual trends in neonatal mortality suggests that underreporting of deaths is more severe closer to the truncation date in the TBHs (Fig. 7). In Angola, the two surveys provide fairly low estimates, but there is a clear drop in levels of NMR in the 2 years directly following the truncation date of the 2006 MIS that included a FBH. In the 2015 TBH survey in Mozambique, the point estimate closest to the truncation date is 40% lower than the estimate closest to the survey date.

### Completeness of data on dates of birth and death

The reported dates of birth are remarkably complete for recent births. The proportion of surviving children with complete information on dates of birth was higher than 99.9% in half of the FBH surveys, and higher than 99.2% in half of the TBH surveys. Despite these high levels of completeness, there were significant differences across types of questionnaires. For example, in the 2016 MIS in Madagascar, dates of birth were complete for 94.3% of children, against 99.9% for recent births reported in the 2008 DHS. In 27 of the 45 survey pairs, completeness of information was significantly higher in FBH than in TBH surveys, whereas the reverse was true in only four survey pairs (Additional file 1: Fig. S5). For deceased children, there is more hesitation about the date of birth; the information is complete in 97.1% in FBH surveys against 93.7% in the paired TBH surveys. Again, completeness was significantly higher in FBHs in 19 pairs of surveys, whereas the reverse is true in only 5 pairs. The TBH surveys thus appear to be less effective at collecting complete data on children’s birth dates than the surveys to which they were paired. This could point to less probing in the TBH surveys if less emphasis was placed on this part of the questionnaire, but it is also possible that the introduction of a truncation causes confusion, especially when reporting on children in reverse order of birth.

We evaluated the quality of reporting of ages at death in surveys conducted in Angola and Mozambique and observed that the proportion of child deaths (aged 1–4 years) for whom the age at death was exactly 1 year (or 1 year without additional information on the month) was respectively 3.5 and 2.3 times higher in TBH surveys than in the paired FBH surveys. Because age at death should be reported in months up to 23 months, data quality on ages at death is much lower in these two TBH surveys (Additional file 1: Table S1). Some of these deaths could be infant deaths reported as having occurred at exact age 1, which may partly explain the downward bias in infant mortality rates in these two surveys.

### Associations with characteristics of the women interviewed and of the interviewers

When considering each survey separately, characteristics of interviewers seem to be associated in several surveys with the reported number of children ever born or deceased, or, the proportion of births and deaths in the reference period (Additional file 1: Figs. S6, S7). However, using the pooled dataset, we find few significant associations in TBHs. The reported number of deceased children is significantly higher when interviewers have experienced a child death (Additional file 1: Table S2), and these interviewers also seem to be associated with more births being reported in the reference period (Additional file 1: Table S3). These interviewers may be able to conduct the interview with more sensitivity and attention, reducing the risk of omissions. In the TBH surveys, we analyzed, about one-third of the interviewers were male. This differs from the standard DHS surveys, where it is typically female interviewers who collect data for the women’s questionnaire. In the FBHs that we exploited, all interviews with women were conducted by women. In a few surveys, we observe some significant associations between the indicators measured and the sex of the interviewers, but these associations are not significant in the pooled TBH dataset (Additional file 1: Tables S2, S3).

Quite surprisingly, the associations with interviewer characteristics appear to be more pronounced in FBH surveys: a lower level of education among interviewers results in fewer births and deaths being reported in the summary birth histories and a lower proportion of deaths in the reference period. Interviewers who have lost a child themselves record more births and deaths in total, but a smaller proportion of these births and deaths are reported in the reference period (presumably because the additional births they record refer to a more distant past). Finally, interviewers in FBH surveys who have prior experience with DHS can capture more deaths in the summary birth histories. These results are intriguing and require further analysis, but most importantly they suggest that interviewer characteristics cannot explain the differences observed between TBH and FBH surveys.

By contrast, the data quality markers in TBHs correlate with respondent attributes. Underreporting of recent births in TBHs is more pronounced among mothers with no education or only primary education (compared to those with secondary or higher education), and in rural (compared to urban) settings (Additional file 1: Fig. S9). Differences across survey instruments are also larger for women with lower educational attainment and those living in rural areas when considering the proportion of deaths reported in the reference period (Additional file 1: Fig. S10).

### Truncation bias

We now examine the selection biases that may arise from truncation. As a reminder, with TBHs, we only have information on the most recent births, while FBHs provide us with deaths and exposure time for the full age range 0–5 over desired reference periods. In reference to Fig. 1b, we are missing the triangle noted *bcd* in TBHs. We consider three implications of this. First, there could be differences in the composition by birth order of the children that are reported. With the 2019–20 DHS conducted in Liberia, we computed the birth-order composition of person-years lived below age five in the quinquennial interval prior to data collection. We present these values based on all children in Fig. 8a (blue curve). This distribution is almost identical when computed only for children born after the truncation date (red curve), i.e., in the area noted *abdef* in the Lexis diagram. This similarity is observed in all the surveys examined in this study. Differences in birth rank composition are therefore unlikely to affect the estimates. The second implication is that mortality for children aged 1–4 years can only be fully observed in the most recent years. Hence, the truncation introduces a downward bias when estimating under-five mortality for the period 0–5 years, due to the mortality decline. In Fig. 8b, we present on the *x*-axis the under-five mortality rate estimated from the 31 different FBH surveys in our study by retaining all children who contribute person-years or deaths during this reference period, alongside rates obtained for that same reference period by selecting only those children who would appear in a TBH on the *y*-axis. The differences are often not significant: the estimate from the full sample is contained in 95% intervals computed from the truncated sample in 28 surveys out of 31. Yet, there is a systematic underestimation, of the order of 4% on average, when restricting the calculation to the most recent births, solely because of this truncation effect. The bias will logically increase when the duration of the reference period is reduced. There is also more uncertainty in the resulting estimates: the coefficient of variation (the ratio of the standard error to the estimate) of U5MR based only on the most recent births is 12% higher on average than when based on the full sample. This problem can be offset by increasing the sample size, but in this case, the time and cost savings offered by using TBHs are also reduced.

## Discussion

Reliable and up-to-date estimates of mortality and fertility remain a pressing need to support program planning and evaluate progress toward development goals, including the ability to track short-term changes. In countries lacking comprehensive systems of birth and death registration, FBHs collected in large-scale survey programs such as the DHS remain key data sources but they might not be the most cost-effective approach. In addition, FBHs may not be suitable whenever there is a need for a shorter instrument that can be deployed in rapid turnaround surveys (e.g., mobile phone surveys) that are cheaper and remain feasible in the situations where face-to-face data collection is hindered due to an epidemic outbreak or other crisis situation. TBHs seem a promising alternative. However, this study provides evidence of extensive underreporting of recent births and recent deaths in 32 surveys that used TBHs, especially in the year immediately following the truncation date. We also observed that a greater proportion of dates of birth had to be imputed in the truncated birth histories. Data quality on ages at death was significantly lower in Angola in 2006–7 and Mozambique in 2015 than in the paired FBH surveys, and the mortality levels were implausibly low in these two surveys.

In our analysis of interviewer characteristics, we found no compelling evidence that differences in data quality between FBHs and TBHs can be ascribed to the interviewers themselves, whereas data quality markers correlate with respondent attributes. This suggests that TBHs are less well understood by respondents, although it is also possible that less educated and rural women are insufficiently assertive to correct an enumerator who might be leading the interview in a certain direction. With the available data, there is no way to establish whether misreporting errors made by respondents were caused by confusion introduced by the truncation itself, or, were associated with changing the order of births being reported in birth histories. There is some limited literature on the effect of changing this order on misreporting errors. Becker and Mahmud conducted a validation study in Matlab, Bangladesh, and compared error patterns between ‘backward’ and ‘forward’ pregnancy histories [33]. They observed that omissions of births and errors in dates of birth were more frequent when using the forward questionnaire. Potter also argued that birth history questionnaires should begin with the most recent, rather than the first birth [34]. This recommendation is based on the assumption that women tend to telescope earlier births toward the date of the interview, and that recent births are more accurately reported. As a result, recent fertility estimates are unbiased, but there is a downward bias in fertility estimates for the distant past, and an upward bias in the intervening period (5–15 years before the survey). This pattern of error is more likely to occur when birth histories are collected from the first birth to the present, as in FBH. Our analysis suggests, however, that collecting birth histories in reverse chronological order can also result in severe bias when truncating these birth histories. Since children are listed from the most recent birth, some women could assume that no other child could be included in the reference period once a birth had been dated close enough to the truncation date. Because birth intervals are much shorter when the preceding child has died [35], deceased children are more likely to have been born closer to this penultimate birth and to be omitted from the reference period.

Our study has several limitations. First, the observations we made are valid for MIS, but may not be generalizable to other survey programs. It is possible that the MIS surveys are affected by a greater omission of children than other surveys that used TBHs because the measurement of under-five mortality may not have been a priority for MIS as it is the case for standard DHS. The training of interviewers may have been less comprehensive for this part of the questionnaire, simply because questions on ages at death were not asked in many MIS. The training period for MIS surveys is also usually shorter than for standard DHS. To give just an example, for the 2018–2019 MIS in Uganda, the main training of interviewers lasted 18 days, while the main training for the 2016 DHS lasted a month. (The DHS questionnaire was also longer with additional modules including maternal mortality and domestic violence.) Nevertheless, we observed significant differences between TBHs and FBHs even in cases where the two surveys being compared were MIS surveys, so it does seem that some of the errors uncovered here are attributable to the truncation. Our results on underreporting of recent deaths are also consistent with Hill’s work on RHS surveys in Mongolia [21].

Second, we are comparing surveys conducted several years apart, so the variations observed could reflect differences in the composition of the samples, and actual trends in mortality and fertility. To reduce this bias, we worked on 45 pairs of surveys, included FBH surveys conducted before and after the TBH surveys, and referred to model-based estimates of fertility and mortality from UN IGME and the WPP.

Third, we were only able to estimate age-specific mortality in Angola and Mozambique, as other MIS surveys did not collect information on ages at death.

Despite these limitations, our analysis suggests that great caution should be exercised when truncating birth histories. It appears that the time saved by using this shorter instrument (restricting the birth history to about one-third of births) does not offset the risks of introducing additional data errors, nor the reduced ability to document trends and age patterns. If timing and resources constraints dictate the use of TBHs, we recommend setting the truncation date not too close to the survey date (e.g., 10 years before the survey), probing and recording the date of birth of the last birth before the truncation date, and implementing continuous monitoring of certain data quality indicators during collection (such as the proportion of children reported in the reference period). Questions on ages at death should also be systematically collected to better measure mortality and allow for conducting additional data quality checks. Further studies should be conducted to confirm that this instrument confuses respondents. For example, additional work could focus on TBHs conducted outside of the DHS program and leverage Reproductive Health Surveys or country-specific surveys. Validation studies using randomized controlled trials in which different survey instruments are administrated to subgroups of the population being interviewed would be useful [7, 36]. Innovative modes of collecting data on recent births should also be tested, such as adding recall cues or probing questions (e.g., on the birth intervals between siblings), using major events as anchors for the start of the reference period, or letting respondents list the children in the order they come to mind before rearranging them by birth order.

Given the high burden of under-five mortality globally (5 million deaths in 2021 [31]) and the centrality of fertility trends in shaping population dynamics, improving the collection of reproductive histories should be a key priority for epidemiologists and demographers. At the same time, improvements to survey instruments should not distract us from an even more pertinent goal: strengthening vital registration systems to enable continuous monitoring of demographic events and honoring the right to be counted.

## Supplementary Information

**Additional file 1. **Supplementary Tables and Figures.

## Data Availability

All data are publicly available through www.dhsprogram.com. We used the survey and demogsurv packages of the R statistical software. See https://github.com/mrc-ide/demogsurv for demogsurv. All R scripts are available upon request.

## Abbreviations

CRVS: Civil Registration and Vital Statistics Systems
DHS: Demographic and Health Surveys
FBH: Full birth histories
FBH: Full pregnancy histories
MIS: Malaria Indicator Surveys
RHS: Reproductive Health Surveys
SBH: Summary birth histories
TBH: Truncated birth histories

## Footnotes

1 A few children born more than 6 years before data collection were reported in birth histories limited to a fixed 6-year reference period, but these represent less than 4% of births and have been removed from the following analysis. In the 2006 MIS Senegal, no birth is reported in the period 5–6 years before the survey, so we truncated the FBHs accordingly.

## References

[1] Mikkelsen L, Phillips DE, AbouZahr C, Setel PW, de Savigny D, Lozano R, Lopez AD (2015). A global assessment of civil registration and vital statistics systems: monitoring data quality and progress. Lancet.

[2] Sharrow D, Hug L, You D, Alkema L, Black R, Cousens S, Croft T, Gaigbe-Togbe V, Gerland P, Guillot M, Hill K, Masquelier B, Mathers C, Pedersen J, Strong KL, Suzuki E, Wakefield J, Walker N (2022). Global, regional, and national trends in under-5 mortality between 1990 and 2019 with scenario-based projections until 2030: a systematic analysis by the UN inter-agency group for child mortality estimation. Lancet Glob Health.

[3] Hill K, Moultrie T, Dorrington R, Hill A, Hill K, Timæus I, Zaba B (2013). Indirect estimation of child mortality. Tools for demographic estimation, Chap. 16.

[4] Moultrie T, Dorrington R, Hill A, Hill K, Timæus I, Zaba B (2013). Tools for Demographic Estimation.

[5] Silva R (2012). Child mortality estimation: consistency of under-five mortality rate estimates using full birth histories and summary birth histories. PLoS Med.

[6] Akuze J, Cousens S, Lawn JE, Waiswa P, Gordeev VS, Arnold F, Croft T, Baschieri A, Blencowe H (2021). Four decades of measuring stillbirths and neonatal deaths in Demographic and Health Surveys: historical review. Popul Health Metrics.

[7] Espeut D, Becker S (2015). The validity of birth and pregnancy histories in rural Bangladesh. J Health Popul Nutr.

[8] Pullum TW, Becker S. Evidence of omission and displacement in DHS birth histories. Technical report, Rockville (2014). Available at http://dhsprogram.com/pubs/pdf/MR11/MR11.pdf.

[9] Akuze J, Blencowe H, Waiswa P, Baschieri A, Gordeev VS, Kwesiga D, Fisker AB, Thysen SM, Rodrigues A, Biks GA, Abebe SM, Gelaye KA, Mengistu MY, Geremew BM, Delele TG, Tesega AK, Yitayew TA, Kasasa S, Galiwango E, Natukwatsa D, Kajungu D, Enuameh YA, Nettey OE, Dzabeng F, Amenga-Etego S, Newton SK, Tawiah C, Asante KP, Owusu-Agyei S, Alam N, Haider MM, Imam A, Mahmud K, Cousens S, Lawn JE, Ayele TA, Bisetegn TB, Delwar N, Gezie LD, Gyezaho C, Kaija J, Machiyama K, Manu G, Manu AA, Martins JS, Melese T, Alam SS, Nareeba T, Hardy VP, Zandoh C, Arnold F, Byass P, Croft T, Herbst K, Kishor SK, Serbanescu F (2020). Randomised comparison of two household survey modules for measuring stillbirths and neonatal deaths in five countries: the every newborn-INDEPTH study. Lancet Glob Health.

[10] Adjiwanou V, Alam N, Alkema L, Asiki G, Bawah A, Béguy D, Cetorelli V, Dube A, Feehan D, Fisker AB, Gage A, Garcia J, Gerland P, Guillot M, Gupta A, Haider MM, Helleringer S, Jasseh M, Kabudula C, LeGrand T, Masquelier B, Menashe-Oren A, Moultrie TA, Queiroz BL, Reniers G, Soura A, Timæus IM, You D (2020). Measuring excess mortality during the COVID-19 pandemic in low- and lower-middle income countries: the need for mobile phone surveys. Center Open Sci.

[11] Bryce J, RMM Working Group (2016). Real-time monitoring of under-five mortality: a vision tempered by reality. PLoS Med.

[12] Hill K, Brady E, Zimmerman L, Montana L, Silva R, Amouzou A (2015). Monitoring change in child mortality through household surveys. PLoS One.

[13] Merdad L, Hill K, Levin M (2016). Data on survival of recent births as a source of child mortality estimates in the developing world: an assessment of census data. Popul Stud.

[14] Becker SR, Thornton JN, Holder W (1993). Infant and child mortality estimates in two counties of Liberia: 1984. Int J Epidemiol.

[15] Zimmerman L, Olson H, Tsui A, Radloff S, PMA2020 Principal Investigators Group (2017). PMA2020: rapid turn-around survey data to monitor family planning service and practice in ten countries. Stud Fam Plan.

[16] Masquelier B, Hug L, Sharrow D, You D, Hogan D, Hill K, Liu J, Pedersen J, Alkema L (2018). Global, regional, and national mortality trends in older children and young adolescents (5–14 years) from 1990 to 2016: an analysis of empirical data. Lancet Glob Health.

[17] Schoumaker B. Stalls and reversals in fertility transition in Sub-Saharan Africa. Real or spurious? Working paper SPED 30, Louvain-la-Neuve (2009)

[18] Howell EM, Holla N, Waidmann T (2016). Being the younger child in a large African family: a study of birth order as a risk factor for poor health using the Demographic and Health Surveys for 18 countries. BMC Nutr.

[19] Goldman N, Moreno L, Westoff CF. Peru experimental study, an evaluation of fertility and child health information. Technical report, Princeton (1989). Available at http://dhsprogram.com/pubs/pdf/FR32/FR32.pdf.

[20] Westoff CE, Goldman N, Moreno L. Dominican republic experimental study. Technical report, Columbia (1990). http://dhsprogram.com/pubs/pdf/FR11/FR11.pdf.

[21] Hill K, Moultrie T, Dorrington R, Hill A, Hill K, Timæus I, Zaba B (2013). Direct estimation of child mortality from birth histories. Tools for demographic estimation, Chap. 16.

[22] Hallett T, Gregson S, Kurwa F, Garnett G, Dube S, Chawira G, Mason P, Nyamukapa C (2010). Measuring and correcting biased child mortality statistics in countries with generalized epidemics of HIV infection. Bull World Health Organ.

[23] Walker N, Hill K, Zhao F (2012). Child mortality estimation: methods used to adjust for bias due to AIDS in estimating trends in under-five mortality. PLoS Med.

[24] Pullum T, Juan C, Khan N, Staveteig S. The effect of interviewer characteristics on data quality in DHS surveys. Rockville, Technical report; 2018.

[25] Guillot M, Gerland P, Pelletier F, Saabneh A (2012). Child mortality estimation: a global overview of infant and child mortality age patterns in light of new empirical data. PLoS Med.

[26] United Nations: World Population Prospects: The 2022 Revision. United Nations, New York (2022). https://esa.un.org/unpd/wpp/.

[27] Alkema L, Raftery A, Gerland P, Clark SJ, Pelletier F (2012). Estimating trends in the total fertility rate with uncertainty using imperfect data. Demogr Res.

[28] ICF International. Demographic and health survey sampling and household listing manual. : MEASURE DHS, Calverton, 2012.

[29] Lumley T (2004). Analysis of complex survey samples. J Stat Softw.

[30] Pedersen J, Liu J (2012). Child mortality estimation: appropriate time periods for child mortality estimates from full birth histories. PLoS Med.

[31] United Nations Inter-agency Group for Child Mortality Estimation: Levels and Trends in Child Mortality: 2022 Report, New York (2022).

[32] Verhulst A (2016). Child mortality estimation: an assessment of summary birth history methods using microsimulation. Demogr Res.

[33] Becker S, Mahmud S (1984). A validation study of backward and forward pregnancy histories in Matlab, WFS scientific reports.

[34] Potter JE (1977). Problems in using birth-history analysis to estimate trends in fertility. Popul Stud.

[35] Grummer-Strawn LM, Stupp PW, Mei Z, Montgomery M, Cohen B (1998). Effect of a child’s death on birth spacing: a cross-national analysis. From death to birth: mortality decline and reproductive change.

[36] Helleringer S, Pison G, Masquelier B, Kanté A, Douillot L, Duthé G, Sokhna C, Delaunay V (2014). Improving the quality of adult mortality data collected in demographic surveys: validation study of a new siblings’ survival questionnaire in Niakhar, Senegal. PLoS Med.

