# Supplementary material for: An evaluation of truncated birth histories for the rapid measurement of fertility and child survival
Source: Popul Health Metr. 2023 Jul 18;21:8. doi: 10.1186/s12963-023-00307-9 (PMC10354946; doi:10.1186/s12963-023-00307-9)
Supplement: Supplementary file 1 — Additional file 1. Supplementary Tables and Figures. [file 12963_2023_307_MOESM1_ESM.pdf]

ADDITIONAL FILE

An evaluation of truncated birth histories for rapid measurement of fertility and child survival

Bruno Masquelier, Ashira Menashe-Oren, Georges Reniers

Population Health Metrics, 10.1186/s12963-023-00307-9

Table S1 – *Coefficients associated with interviewers' characteristics in multiple linear regression models predicting the reported number of children ever born or deceased*

	Dependent variable:		
	Number of children ever born reported		
	FBH	TBH	FBH & TBH
Male interviewers		0.006	
Interviewers with primary/secondary educ. (vs. higher)	−0.057**	0.023	−0.049*
Interviewers who have experienced a child death	0.358***	−0.015	0.317***
Interviewers who have worked previously for DHS	0.012	−0.028	0.010
Interaction term education × TBH			0.074*
Interaction term child death × TBH			−0.325***
Interaction term work experience × TBH			−0.042
Observations	124,205	103,884	228,089
	Dependent variable:		
	Number of children deceased reported		
	FBH	TBH	FBH & TBH
Male interviewers		−0.004	
Interviewers with primary/secondary educ. (vs. higher)	−0.074***	0.006	−0.073***
Interviewers who have experienced a child death	0.160***	0.032*	0.152***
Interviewers who have worked previously for DHS	0.024***	0.007	0.025***
Interaction term education × TBH			0.080***
Interaction term child death × TBH			−0.120***
Interaction term work experience × TBH			−0.018
Observations	124,205	103,884	228,089

Note: *p<0.1; **p<0.05; ***p<0.01. The models also include as covariates the age group of respondents and their educational attainment, their place of residence, and survey-specific fixed effects.

Table S2 – *Coefficients associated with interviewers' characteristics in multiple logistic regression models predicting the proportion of births and deaths reported in the reference period*

	Dependent variable:		
	Proportion of births in reference period		
	FBH	TBH	FBH & TBH
Male interviewers		0.999	
Interviewers with primary/secondary educ. (vs. higher)	0.998	0.997	0.994
Interviewers who have experienced a child death	0.962*	1.083***	0.988
Interviewers who have worked previously for DHS	0.988	1.026	0.990
Interaction term education × TBH			1.002
Interaction term child death × TBH			1.096**
Interaction term work experience × TBH			1.039*
Observations	358,553	308,478	667,031

	Dependent variable:		
	Proportion of deaths in reference period		
	FBH	TBH	FBH & TBH
Male interviewers		0.907	
Interviewers with primary/secondary educ. (vs. higher)	0.868*	1.090	0.870*
Interviewers who have experienced a child death	0.873*	1.050	0.930
Interviewers who have worked previously for DHS	1.040	0.996	1.034
Interaction term education × TBH			1.255
Interaction term child death × TBH			1.120
Interaction term work experience × TBH			0.977
Observations	44,528	40,694	85,222

Note: *p<0.1; **p<0.05; ***p<0.01. The models also include as covariates the age group of respondents and their educational attainment, their place of residence, and survey-specific fixed effects.

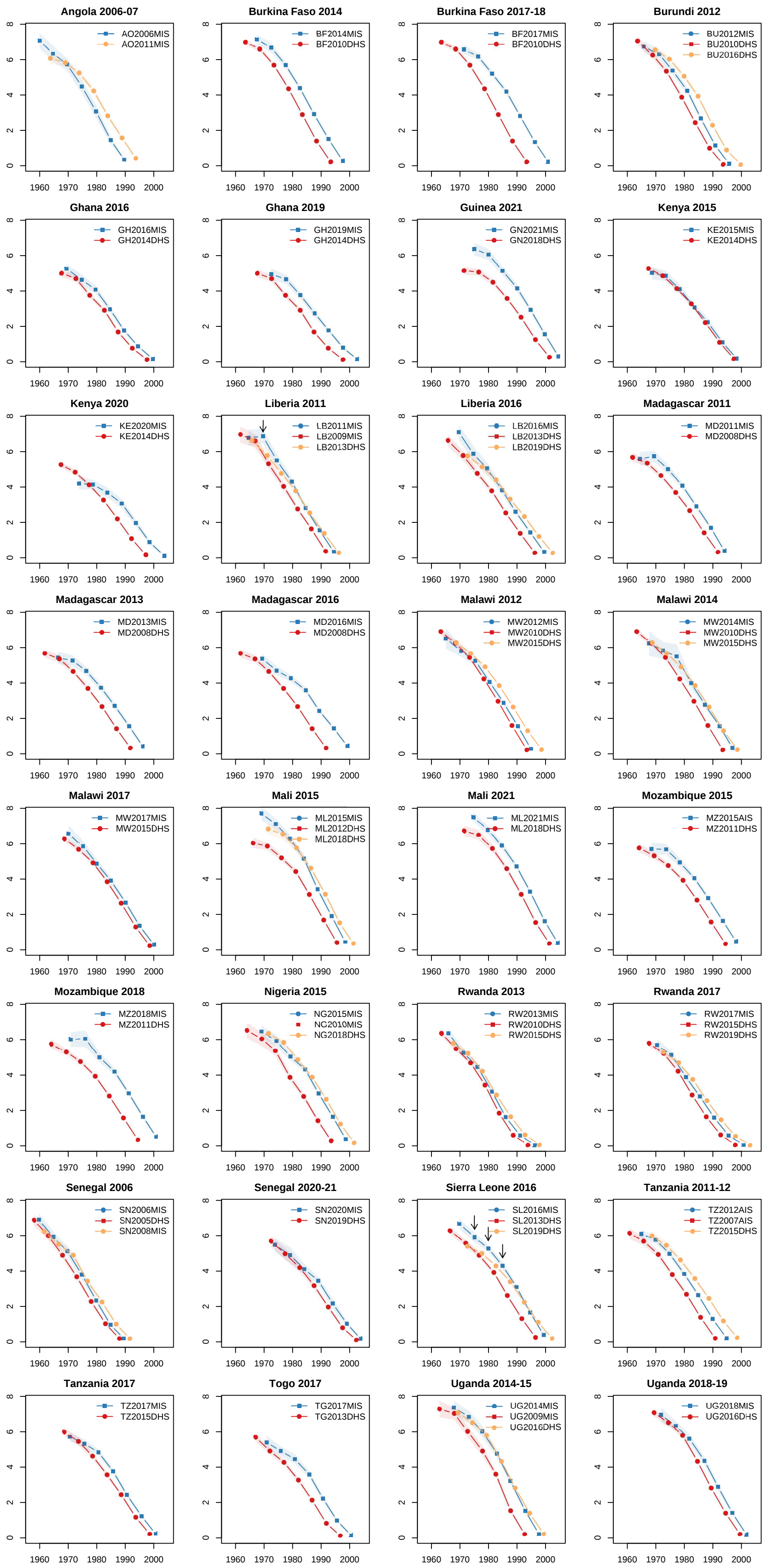

Figure S1 – *Mean number of children ever born reported in TBH surveys and the paired FBH surveys Note: The x-axis refers to the birth cohort of mothers. TBH surveys are identified with squares, FBH surveys with circles. Survey pairs with significant differences pointing to underreporting are identified with arrows.*

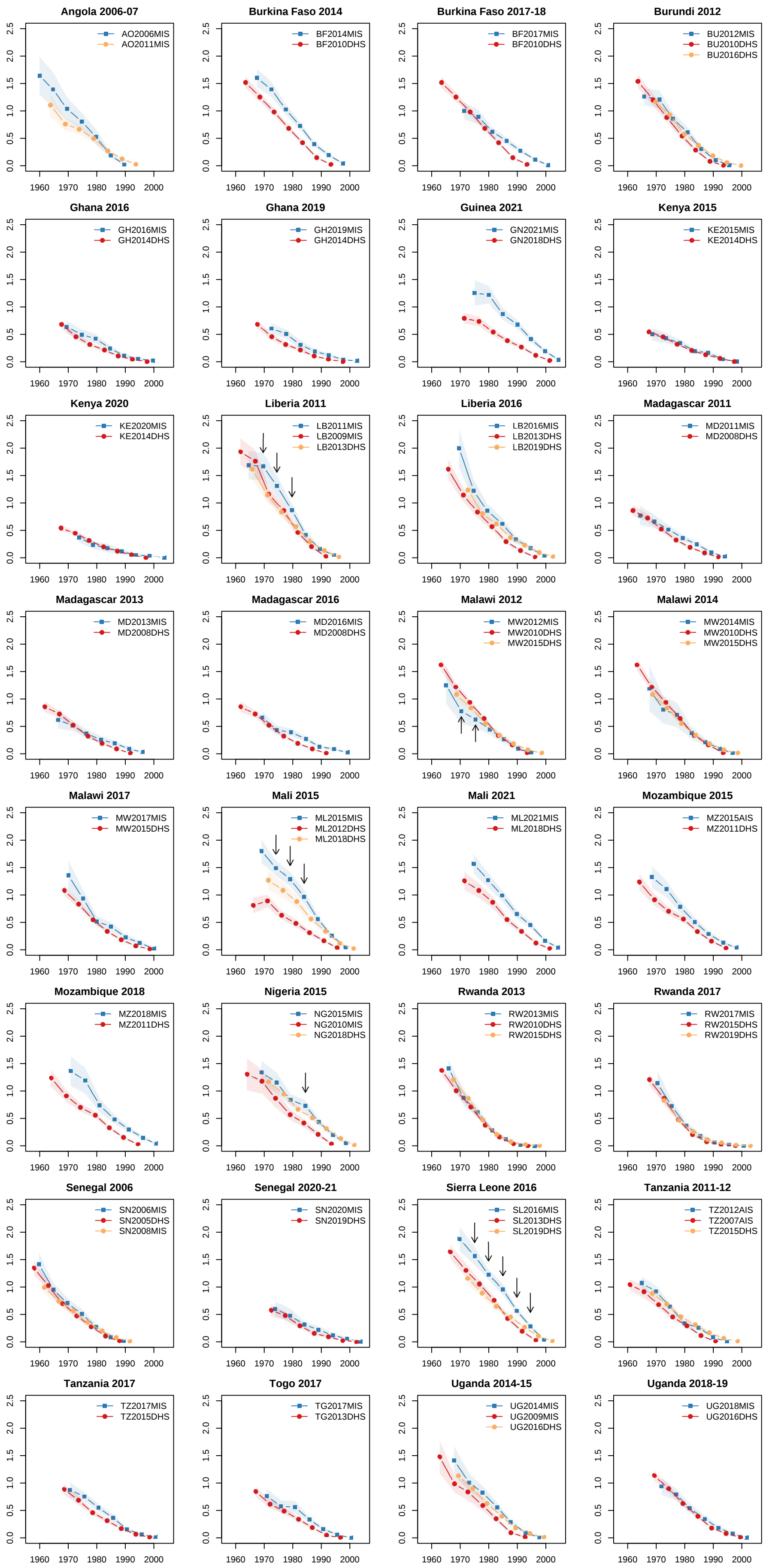

Figure S2 – *Mean number of deceased children reported in TBH surveys and the paired FBH surveys*
Note: The x-axis refers to the birth cohort of mothers. TBH surveys are identified with squares, FBH surveys with circles. Survey pairs with significant differences pointing to underreporting are identified with arrows.

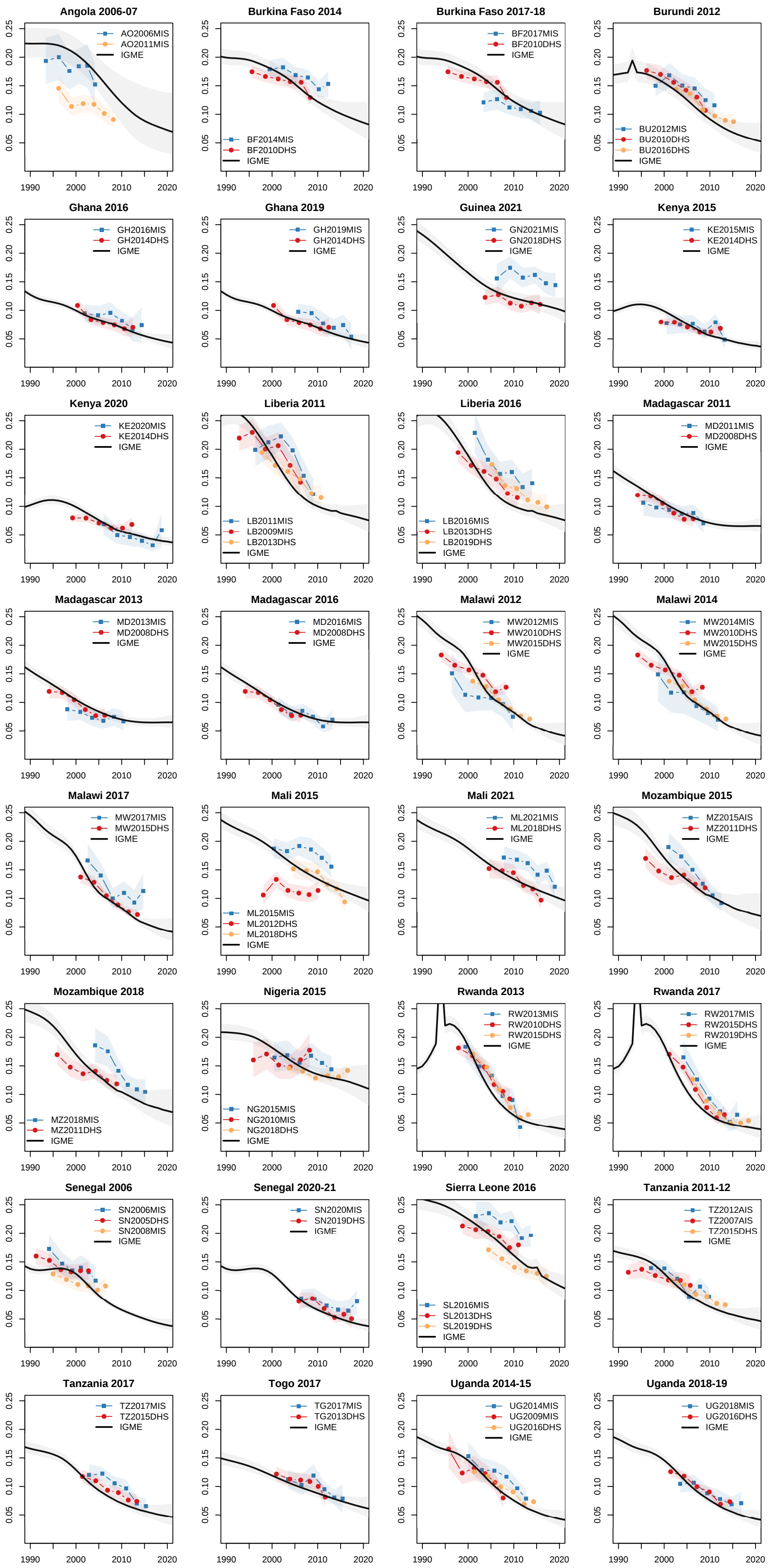

Figure S3 – *Indirect mortality estimates in TBH surveys, the paired FBH surveys and as estimated by the UN IGME (2022)*

Note: TBH surveys are identified with squares, FBH surveys with circles. Shaded areas around survey estimates refer to 95% CI while the shaded areas around IGME estimates refer to 90% CI.

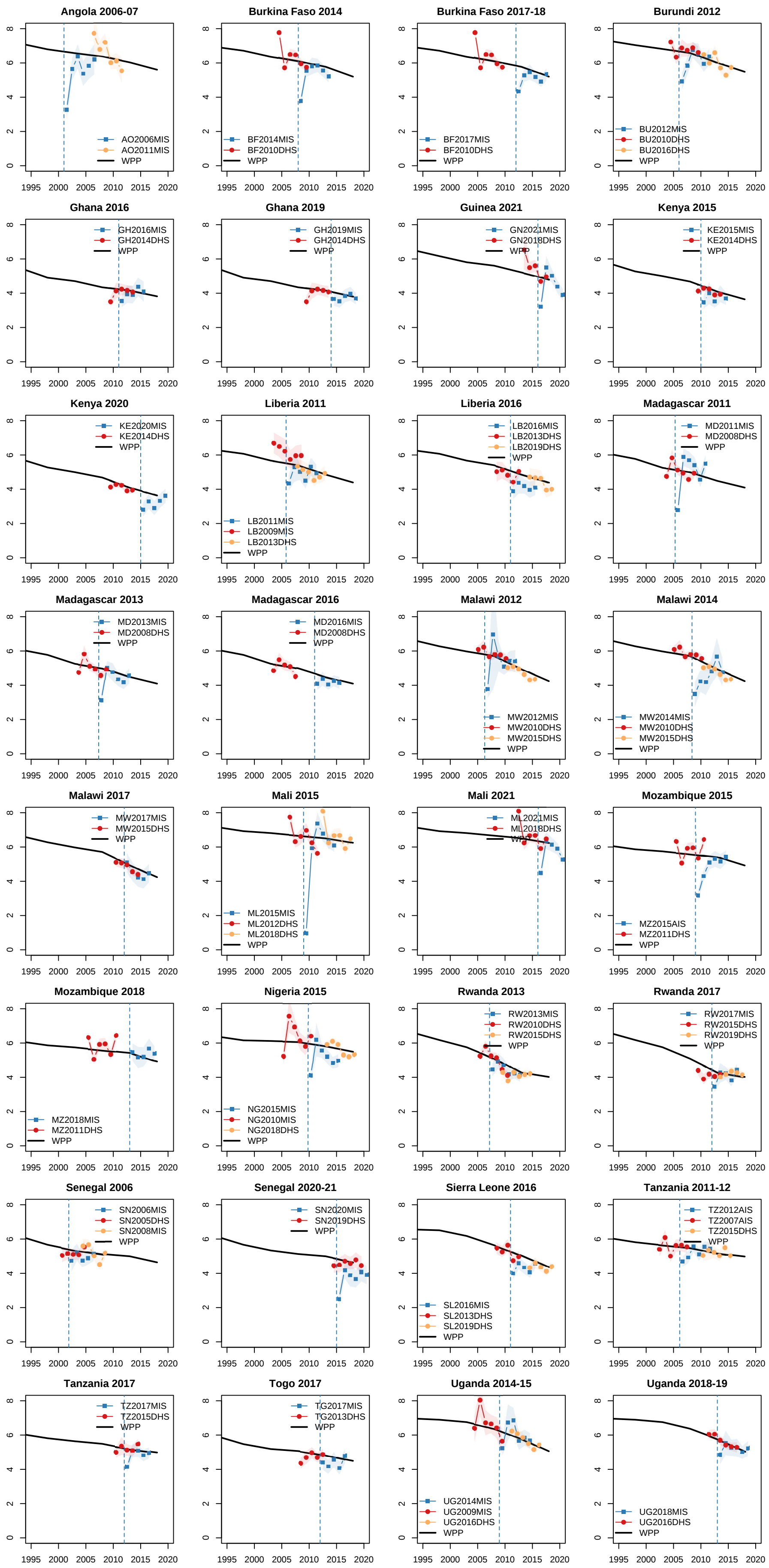

Figure S4 – *Trends in the total fertility rate as estimated from TBH and the paired DHS surveys and as estimated by the WPP 2022. The dashed line refers to the truncation date in the TBH.*

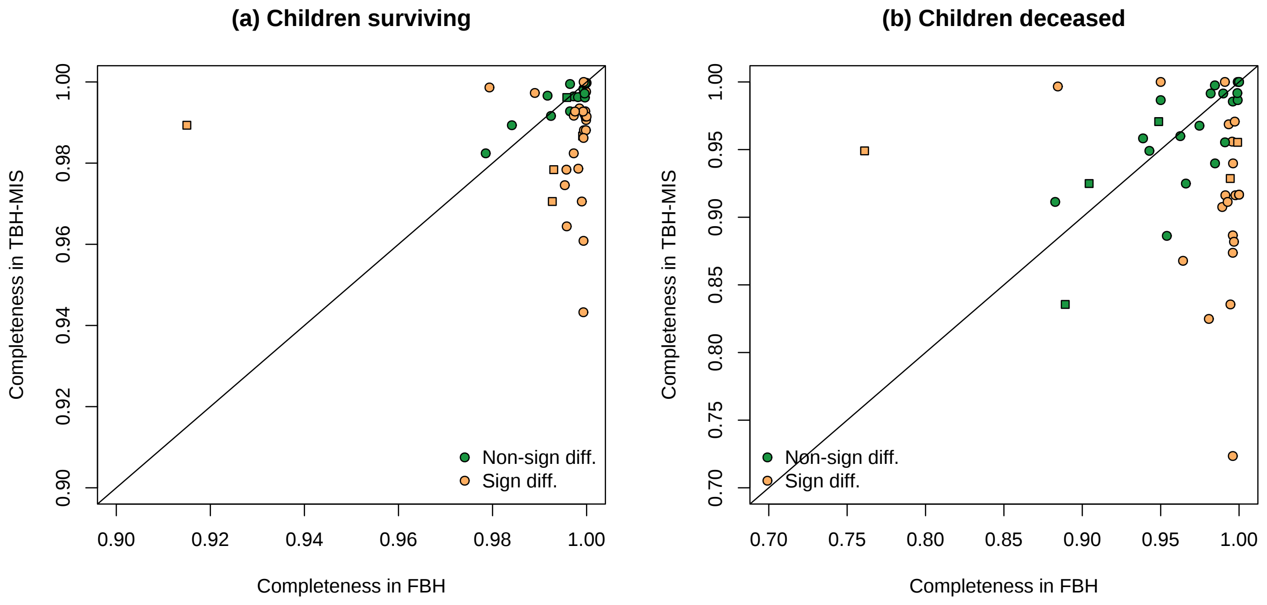

Figure S5 – *Completeness of information dates of birth in TBH and FBH, among children surviving and deceased.*
Note: Survey pairs associating two MIS surveys are identified with squares. These proportions are estimated for recent births falling in the reference period only.

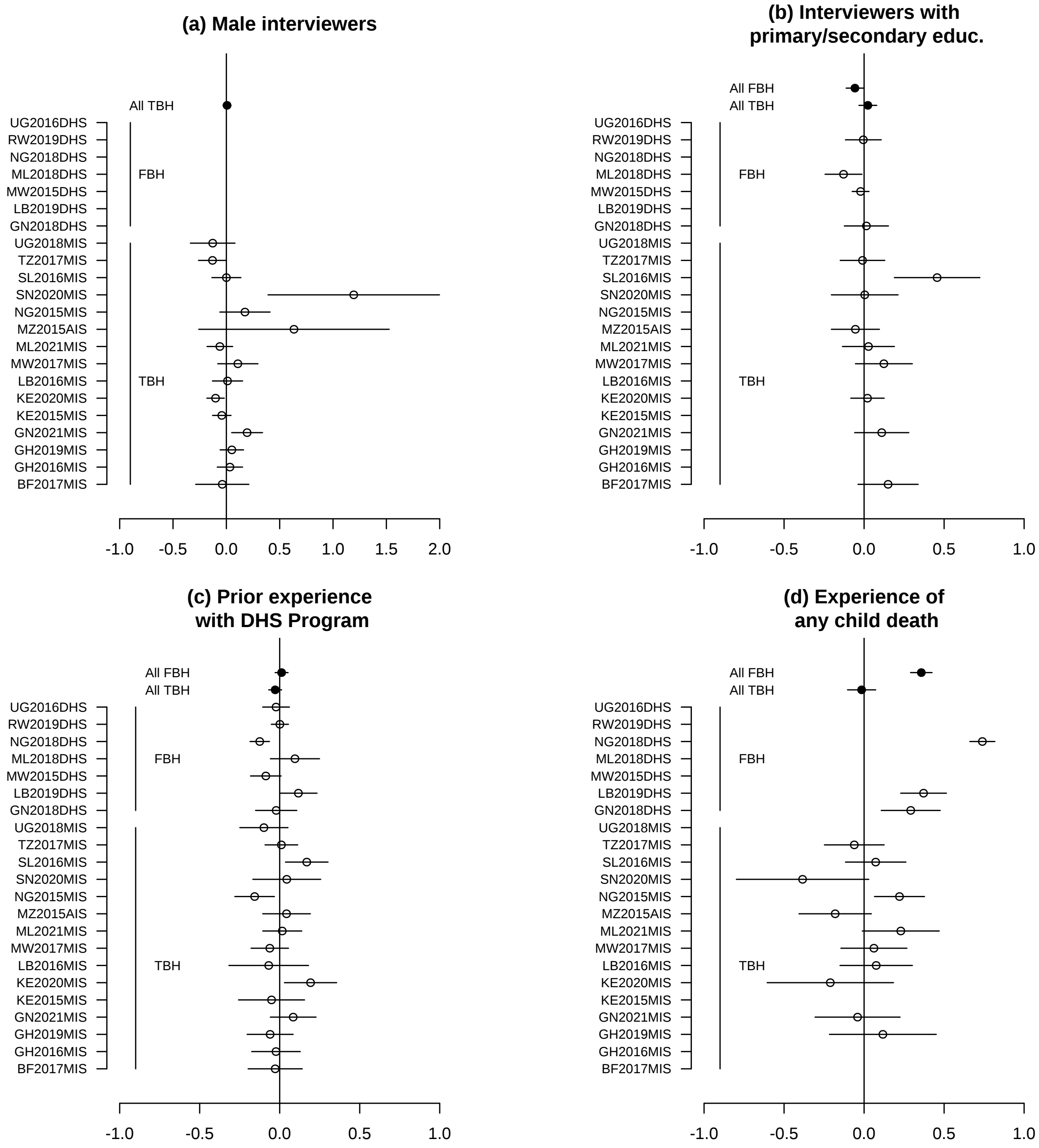

Figure S6 – *Coefficients of multiple linear regression models predicting the reported number of children ever born, by interviewer characteristics*

Note: Coefficients derived from models adjusting for women's age, education and place of residence. The models for pooled datasets also include survey fixed-effects. Coefficients from survey-specific models are not presented when there were less than 5% of interviewers in one category.

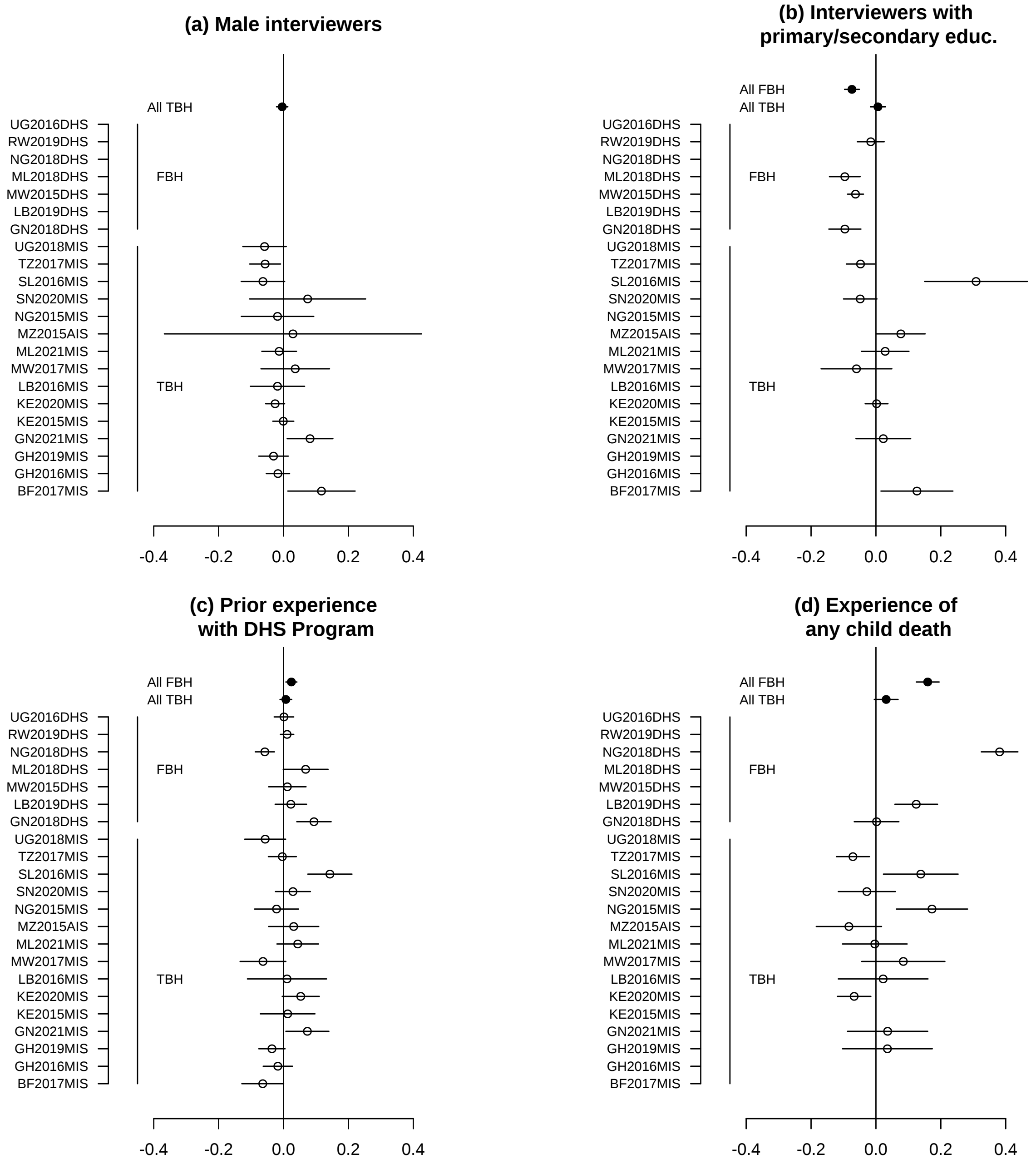

Figure S7 – *Coefficients of multiple linear regression models predicting the reported number of deceased children, by interviewer characteristics*

Note: Coefficients derived from models adjusting for women's age, education, and place of residence. The models for the pooled datasets also include survey fixed-effects. Coefficients from survey-specific models are not presented when there were less than 5% of interviewers in one category.

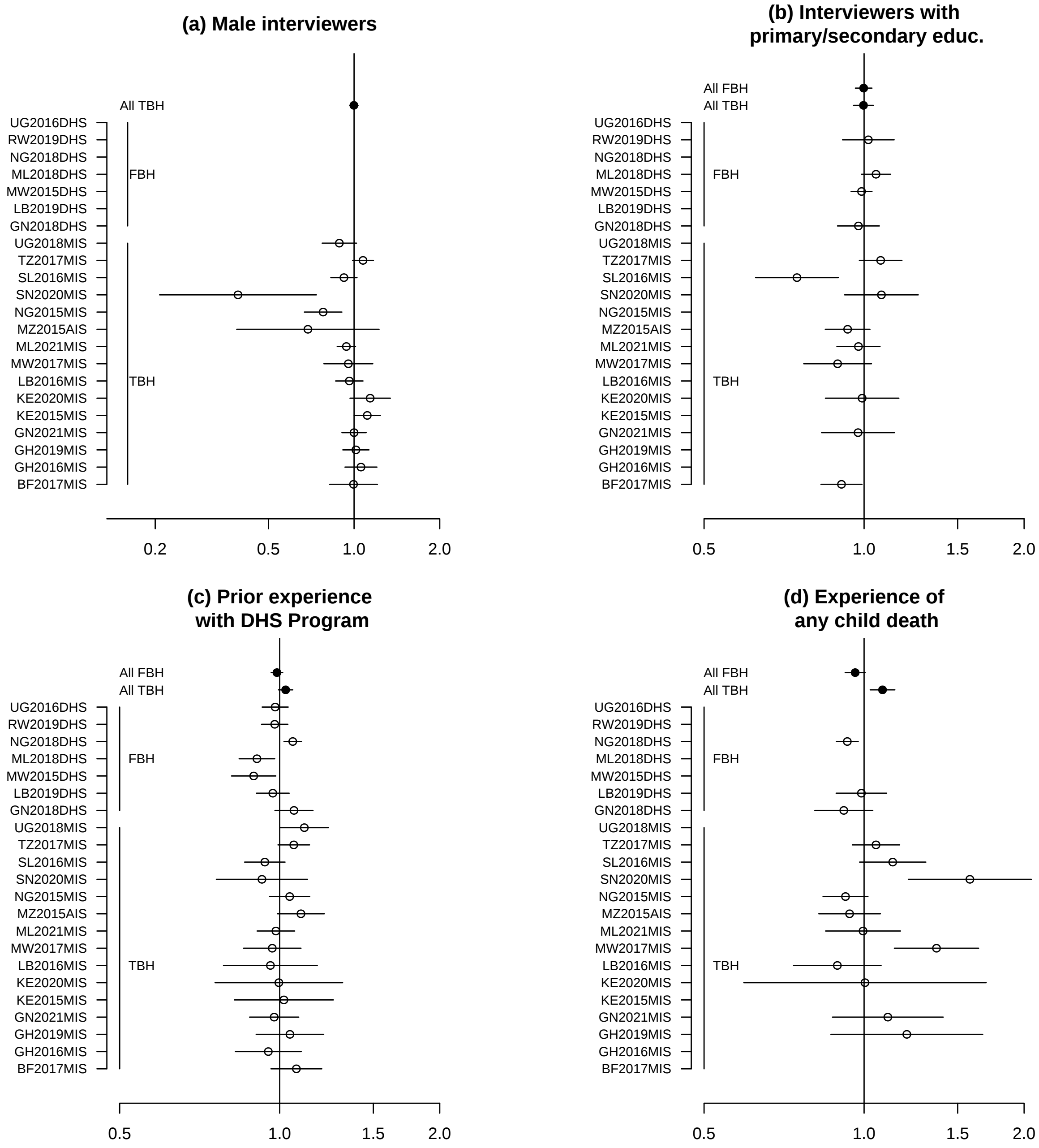

Figure S8 – *Odds ratios from logistic regression models predicting the proportion of births reported in the reference period, by interviewer characteristics*

Note: Odds ratios derived from models adjusting for women's age, education, and place of residence. The models for pooled datasets also include survey fixed-effects. Odds ratios from survey-specific models are not presented when there were less than 5% of interviewers in one category.

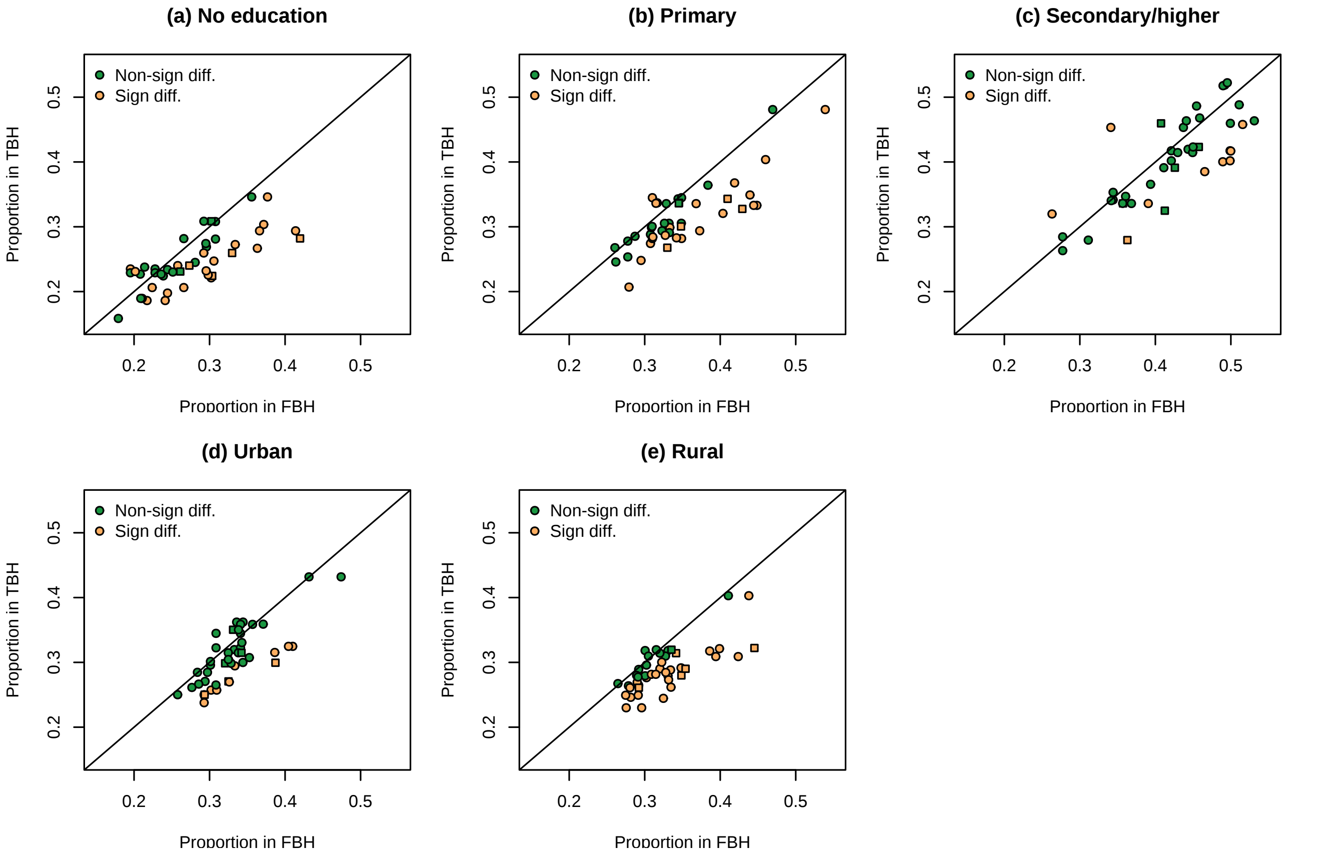

Figure S9 – *Proportions of births reported in the reference period according to the educational level of the mother and to the type of residence of the mother, in TBH and FBH surveys*

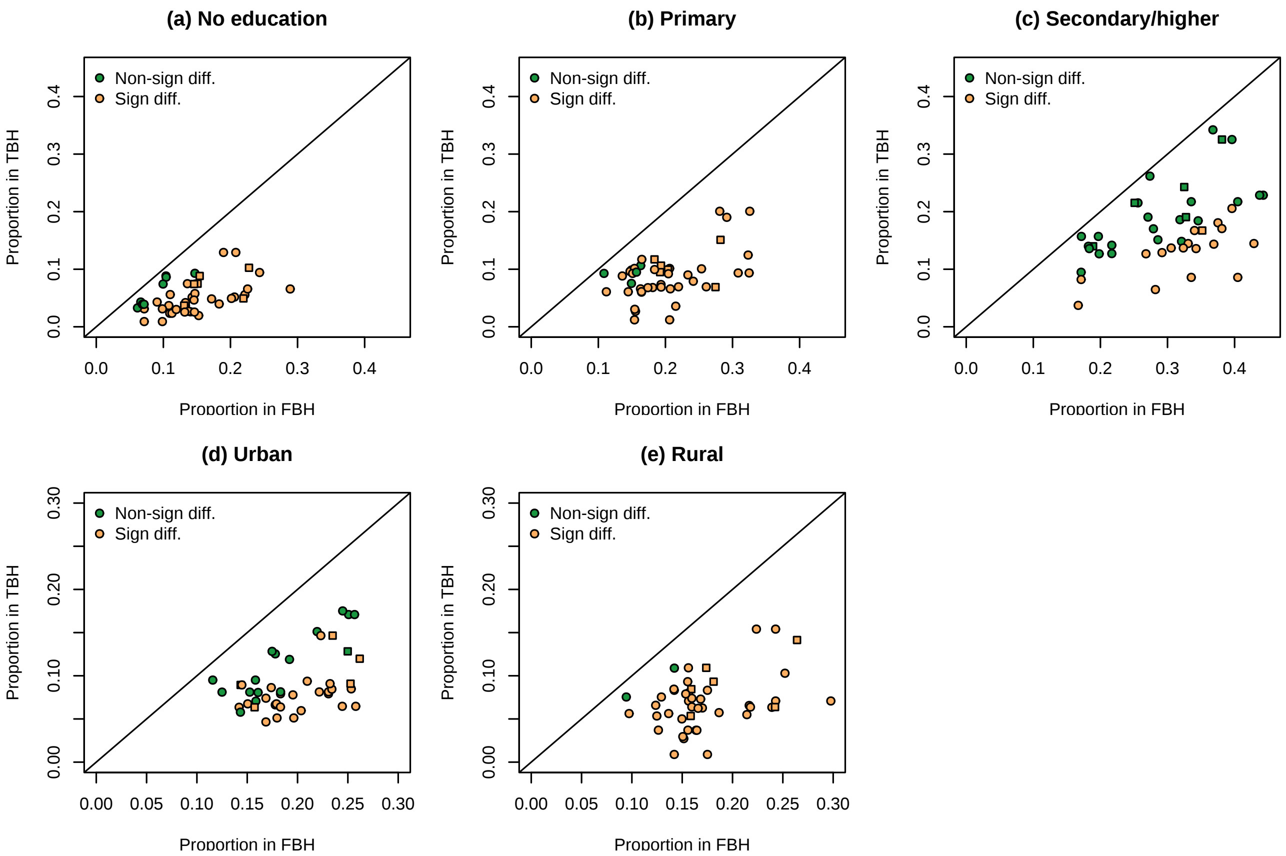

Figure S10 – *Proportions of deaths reported in the reference period according to the educational level of the mother and to the type of residence of the mother, in TBH and FBH surveys*

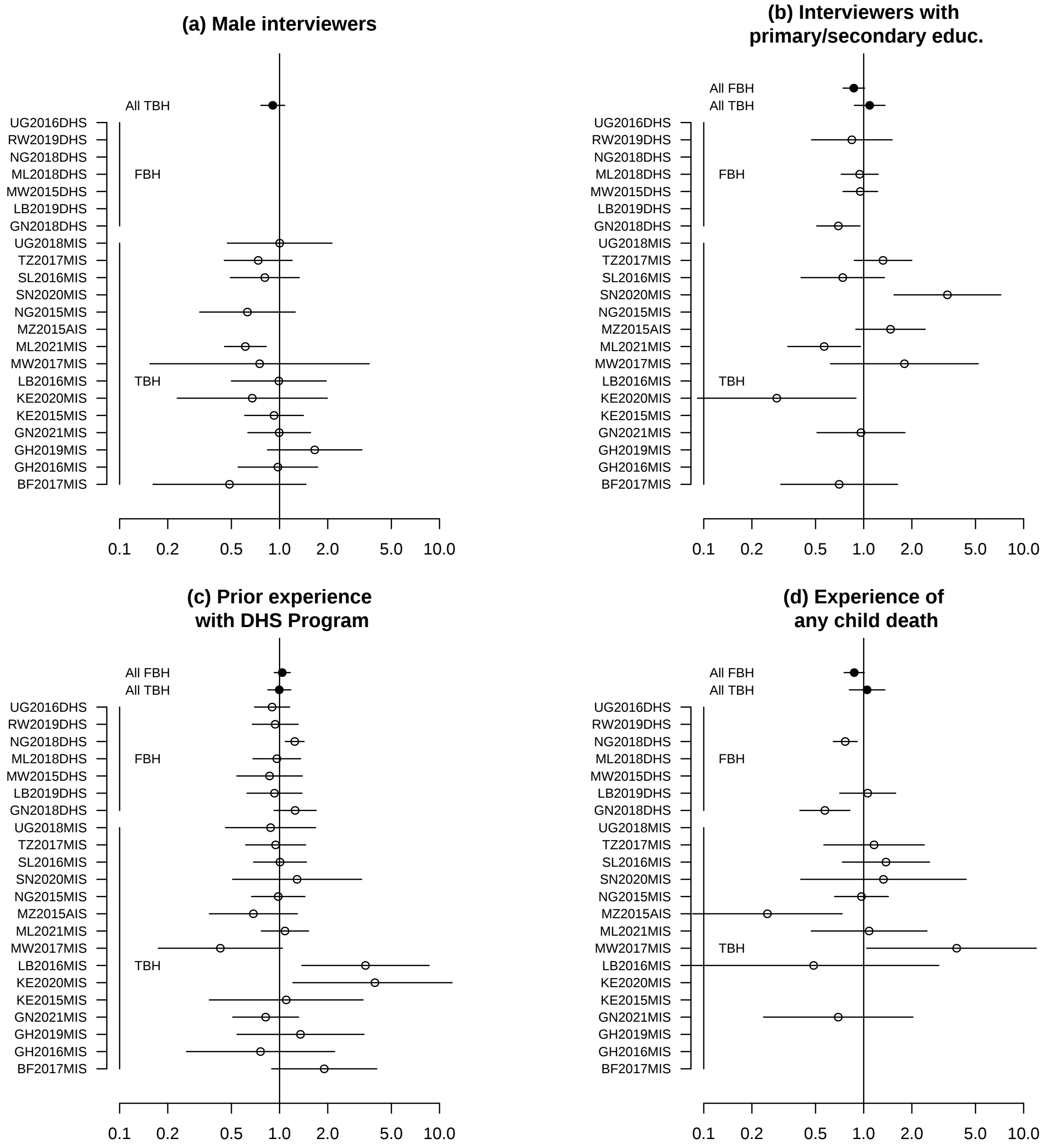

Figure S11 – *Odds ratios from logistic regression models predicting the proportion of deaths reported in the reference period, by interviewer characteristics*

Note: Odds ratios derived from models adjusting for women's age, education and place of residence. The models for the pooled datasets also include survey fixed-effects. Odds ratios from survey-specific models are not presented when there were less than 5% of interviewers in one category.
